# Accelerated Nanocomposite Hydrogel Gelation Times Independent of Gold Nanoparticle Ligand Functionality

**DOI:** 10.1021/acsomega.4c05102

**Published:** 2024-10-14

**Authors:** Brianna Couturier, Gloria Kozak, John Levering, Anna Zini, Meagan B Elinski

**Affiliations:** Department of Chemistry, Hope College, Holland, Michigan 49423, United States

## Abstract

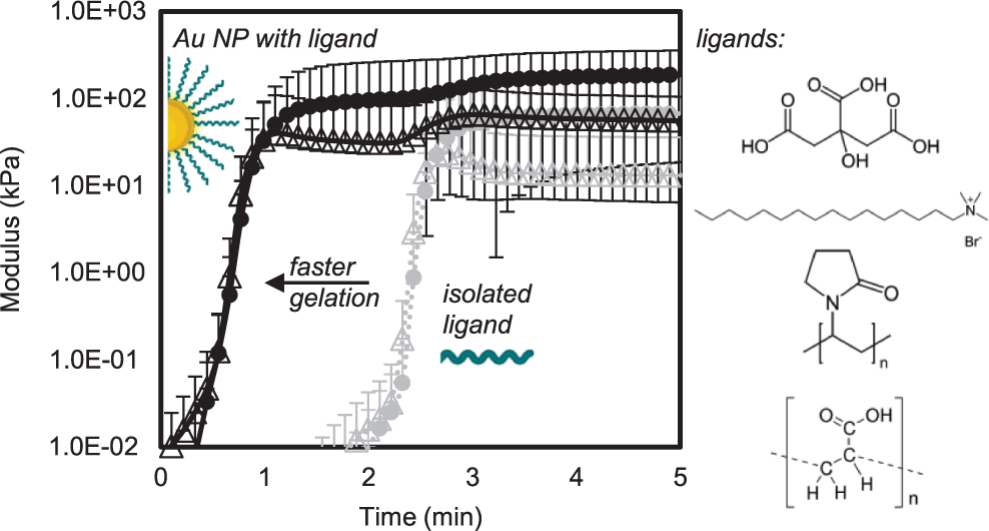

The expansive use of hydrogels in healthcare relies on carefully tuned properties in dynamic environments with predictable behavior, including time sensitive biological systems and biomedical applications. To meet demands in these settings, nanomaterials are often introduced to a hydrogel matrix which simultaneously elevates potential applications while adding complexity to fundamental characteristics. With respect to drug delivery, gold nanoparticles have modifiable surfaces to carry an array of targeted drug treatments. However, different molecules acting as capping ligands possess different chemical structures that can impact gelation times. To understand the influence of capping ligand chemistry on polyacrylamide (PAM) based nanocomposite hydrogel radical gelation time, gold nanoparticle (Au NP) capping ligands were selected to encompass varying functional groups and molecular weights: citrate, cetyltrimethylammonium bromide, polyvinylpyrrolidone, and poly(acrylic acid). Gelation times were quantified as the storage-loss moduli crossover point in rheological time sweeps at constant strain and frequency. The dominating factor for gelation time was the presence of Au NPs, independent of a diverse range of capping ligand structures. The gelation times were also markedly faster than the same capping ligand structures used as stand-alone molecular additives. The accelerated Au NP gelation times, under 2 min, are attributed to the Au NPs acting as a cross-linker, promoting gelation. These results bolster the potential implementation of Au NP nanocomposite hydrogels in time-sensitive biomedical applications as robust drug carriers.

## 1 Introduction

Hydrogels have immense utility in diverse roles including water filtration,^1^ oil production,^2^ flexible electronics and energy storage,^3−5^ and healthcare. In healthcare alone, recent reviews highlight the potential of hydrogels in drug delivery and tissue engineering,^6^ regenerative medicine,^7^ wound dressing, and more.^8,9^ Concurrently, nanoparticles have extensive potential in biomedical applications such as the use of gold nanocrystals for treating neurodegenerative diseases.^10^ As with hydrogels, a multitude of nanoparticle examples are covered in reviews.^11−13^ In particular for drug delivery, the surface chemistry of the nanoparticle dictates their efficacy and selectivity,^14^ as well as interactions with the surrounding environment. Upon entering the body, a protein corona can dynamically form around nanoparticles in biological fluids, requiring careful analysis for predicting nanomedicine safety and efficacy.^15^

Combining nanoparticles and hydrogels (forming nanocomposite hydrogels) builds on the level of complexity, but beneficially provides multifunctional materials for a full suite of biomedical applications (e.g., drug delivery, tissue engineering, wound healing, bioprinting, and biowearable devices).^16^ Gold nanoparticles (Au NPs) have unique physical and optical properties, with tailorable surface chemistry and a strong surface plasmon resonance in the visible and near-infrared regions positioning them for use as passive carriers or active light-responsive vehicles.^17^ Gold-polymer nanocomposites preserve Au NP properties while exhibiting enhanced stability and dispersity, induced responses to stimuli, extended circulation in the bloodstream, and extended retention plus controlled release of active molecules in tissues.^17^

Successful integration of such hydrogel-based technologies with biomedical applications also relies on sensitive timing for various scenarios. In the context of tightly regulated biological systems and medical procedures, hydrogel time parameters such as gelation time determine the biocompatibility, properties, and mode of implementation.^9,18−26^ For example, ionic hydrogels have a tunable gelation time from seconds to minutes and are compatible with injectable delivery, with potential use in filling complex defects via minimally invasive procedures.^20,22^ Injectable preformed hydrogels must further demonstrate dynamic rheological properties,^9,18,19,21,23−25^ such as incorporating dynamic covalent bonds into the backbone or cross-linking moieties.^26^ Another benchmark is the cross-linking method. Physically cross-linked gels may meet the design constraints for injection, while chemically cross-linked gels may be more suitable for ex-vivo gelation followed by implantation.^9,20^ Even with ex-vivo gelation, knowledge of kinetic behavior is necessary in biomedical work.

Focusing on quantifying gelation times for hydrogels and nanocomposite hydrogels sheds light on underlying mechanisms governing gel structures and properties. Gelation times of polyacrylamide (PAM)/polyethylenimine (PEI) hydrogels enhanced with nanosilica decreased gelation times from 27 h (0% nanosilica) to 12 h (1% nanosilica by weight). An accelerated reaction still occurred at an order of magnitude lower concentration, 0.1%, with gelation at 22.5 h. Importantly, the authors note that all else in experimental conditions held constant, gelation time is determined by the cross-linker. With PEI constant, the nanosilica was concluded to act as a cross-linker, with the silano group presumed to cross-link with the amidogen of PAM via a hydrogen bond.^27^ For polymer-based nanocomposite hydrogels derived from polyethylene glycol (PEG) with terminal anthracene groups, silica-coated nanocapsules (10% and 15% v/v, relative to 0%) resulted in faster gelation times due to an acceleration of network connectivity from synergistic polymer-nanocapsule interactions. Exact times were not reported, but rough estimates from the presented data are of a 300 s gel time at 0% nanocapsules decreased to a 200 s gel time at 15% nanocapsules for a 4-arm PEG-anthracene hydrogel.^28^

Nanomaterials acting as cross-linkers was observed in additional systems. When partially hydrolyzed PAM composites incorporated low concentrations (25–100 ppm) of oxide nanoparticles (SiO_2_, Al_2_O_3_, MgO, and Cr_2_O_3_), coupled with improved gel strength there was decreased gelation time from 14 h (0 ppm nanoparticles) to 12 h (100 ppm nanoparticles) due to nanoparticle interactions with the polymer chains, acting as cross-linkers.^29^ Chitosan based injectable hydrogels infused with chitin nanowhiskers (CNW) also exhibited enhanced mechanical properties and shorter gelation times (from 6038 to 25 s for 0% to 5% CNW at 37 °C), with the nanowhiskers functioning as a cross-linker through hydrogen bonding in the gel formation process.^30^

Hydrogel nanoadditives resulting in decreased gelation times, however, is not a guarantee. PAM/PEI gels doped with graphene oxide (GO) nanosheets resulted in increased gelation times with GO content. Estimating values for the impact of GO from Figure 16 in Zhang et al.,^31^ the gelation times increased from 50 h at 0 wt % to 96 h at 0.025 wt %, and further increased to 103 h at 0.21 wt %.^31^ Other additive types providing gelation dynamics insight include a 2-acrylamido-2-methylpropanesulfonic acid (AMPS), N-vinyl-2-pyrrolidone (NVP), acrylamide terpolymer, PEI system with bentonite (clay) filler. In this system, the nonionic functional groups of the acrylamide and NVP polymer chains adsorbed onto the clay particles, reducing available cross-linking sites to react with the PEI, slowing gelation times (11 h at 0 wt % to 15 h at 1.6 wt %).^32^ Similarly, another clay-nanocomposite hydrogel system comprised of partially hydrolyzed polyacrylamide, PEI, and hexadecyltrimethylammonium bromide surfactant-modified clay does not comment on gelation times, but supported the modified clay having greater interactions with the polymer chains through van der Waals interactions.^33^

Metal additives, separate from metal nanoparticles, also impact gelation times. A slow releasing rate of titanium(IV) from a titanium tartrate complex within partially hydrolyzed PAM delayed gelling. No obvious gelation occurred within 25 h at a 1:1.15 molar ratio of titanium/tartaric acid, relative to a 1:0.5 molar ratio exhibiting signs of gelation within 12 h.^34^ A PAM/chromium (VI) hydrogel (reduced to chromium(III) upon mixing) followed Arrhenius behavior, with gelation time decreasing as temperature was increased for a given concentration. Estimated from Figure 4 in Jordan et al.,^35^ gelation times changed from 23 min at 25 °C to <1 min at 80 °C at a concentration of 1724 g/m^3^ of Betz 1160, a cationic polyacrylamide copolymer with 20% positive charge density.^35^

Predicting the gelation times of nanocomposite hydrogels is thus nontrivial, and necessitates careful quantization for potential biomedical applications. There is also a gap in understanding the influence of surface functionalities of nanoparticles on gelation times. This work focuses on gold nanoparticle (Au NP) additives in PAM hydrogels comparing a range of chemical structures to represent different functionalities of a broad scope of therapeutics. Example therapeutic systems include using Au NPs as drug delivery vehicles for methotrexate (MTX) and doxorubicin (DOX) in cancer treatments.^36^ MTX contains ring and linear areas, and amine, amide, and carboxylic acid functional groups. In the preparation of Au-MTX systems, MTX can replace an initial citrate capping ligand.^37^ DOX contains primarily ring structures, with carboxylic acid, amine, alcohol, ether, and ketone functional groups. The anticancer drug oxaliplatin has also been studied for use with Au NP drug delivery.^36^ Other small molecule examples include using thiolated drugs to bind directly to the Au, cetyltrimethylammonium bromide (CTAB) to attach small interfering RNA (siRNA) to Au nanorods for delivery to dopaminergic neuronal (DAN) cells, and functionalization of Au with chitosan for delivering insulin.^36^ Larger polymer structures such as poly ethylene glycol have also been employed, enhancing the drug delivery system’s stability in physiological conditions,^36^ or serving as a spacer between the NPs and drugs.^37^ Polymers such as poly(acrylic acid) (PAA) when coupled with Au nanorods are effective in hyperthermia therapy, and Au NPs with PAA and a mesoporous silica shell enhance fluorescence for cancer diagnostics.^38^ Polyvinylpyrrolidone (PVP) improves stability and retention times of Au NP based imaging diagnostics, but also exhibits higher risk of cytostatic effects, underscoring the need for deepening the understanding of Au NP capping ligands.^39^

Given these examples as just a subset of the wide scope of nanocontaining therapeutics, for the work here selected ligands included monomers vs polymers, different molecular weights, linear vs ring structures, and extent of hydrogen bonding sites. With distinct features for each ligand, we emphasize that the impact on gelation time cuts across an appreciably varying range of ligands. We chose citrate and CTAB as commonly used small molecules, containing carboxylic acid and amine functional groups, respectively, and differing structures with CTAB having a longer (C_16_) linear chain. PVP and PAA were selected as representative polymers, with different chemical functionalities and molecular weights (40 000 g/mol vs 10 000 g/mol, respectively). These chemical structures were further compared as Au NP capping ligands vs isolated, or stand-alone molecular additives. Radical gelation times were measured in a rheometer, using the principle that rheological time sweeps are sensitive to the sol-to-gel transition, marked by the crossover point of the storage (*G*′) and loss (*G*″) moduli.^40^ We expected the gelation time of the Au NP additive nanocomposite hydrogels to be dominated by the surface chemical interactions of the capping ligand with the surrounding matrix, following similar trends to the isolated ligands, tuned by the differing chemical features. Instead, our primary finding is that the presence of Au NPs accelerated PAM gelation time, independent of capping ligand chemical structure, suggesting a robust mechanism for a broad range of potential time-sensitive drug-delivery applications.

## 2 Materials and Methods

### 2.1 Preparation of Polyacrylamide Hydrogels and Composites

Polyacrylamide (PAM) precursor solutions were prepared with 2.25 mL acrylamide, 375 μL N,*N*′-methylenebis(acrylamide), 30 μL ammonium persulfate (APS, 0.1 g/mL), and 3 μL N,N,*N*′,*N*′-tetramethylenediamine (TEMED, 0.1 g/mL) in 375 μL of Milli-Q water (all PAM chemicals were from Sigma-Aldrich; Milli-Q water was from a Thermo Scientific GenPure Pro purification system). The acrylamide, N,*N*′-methylenebis(acrylamide), and water were mixed and sonicated for 20 min. To form a pure polyacrylamide hydrogel for strain and frequency measurements, following sonication the TEMED and APS were added to the acrylamide-based solution forming a net total solution volume of 3033 μL, and poured into a 35 mm × 10 mm (9 mL) Petri dish. The samples were covered and left to soak in water overnight.

**Figure 1:**
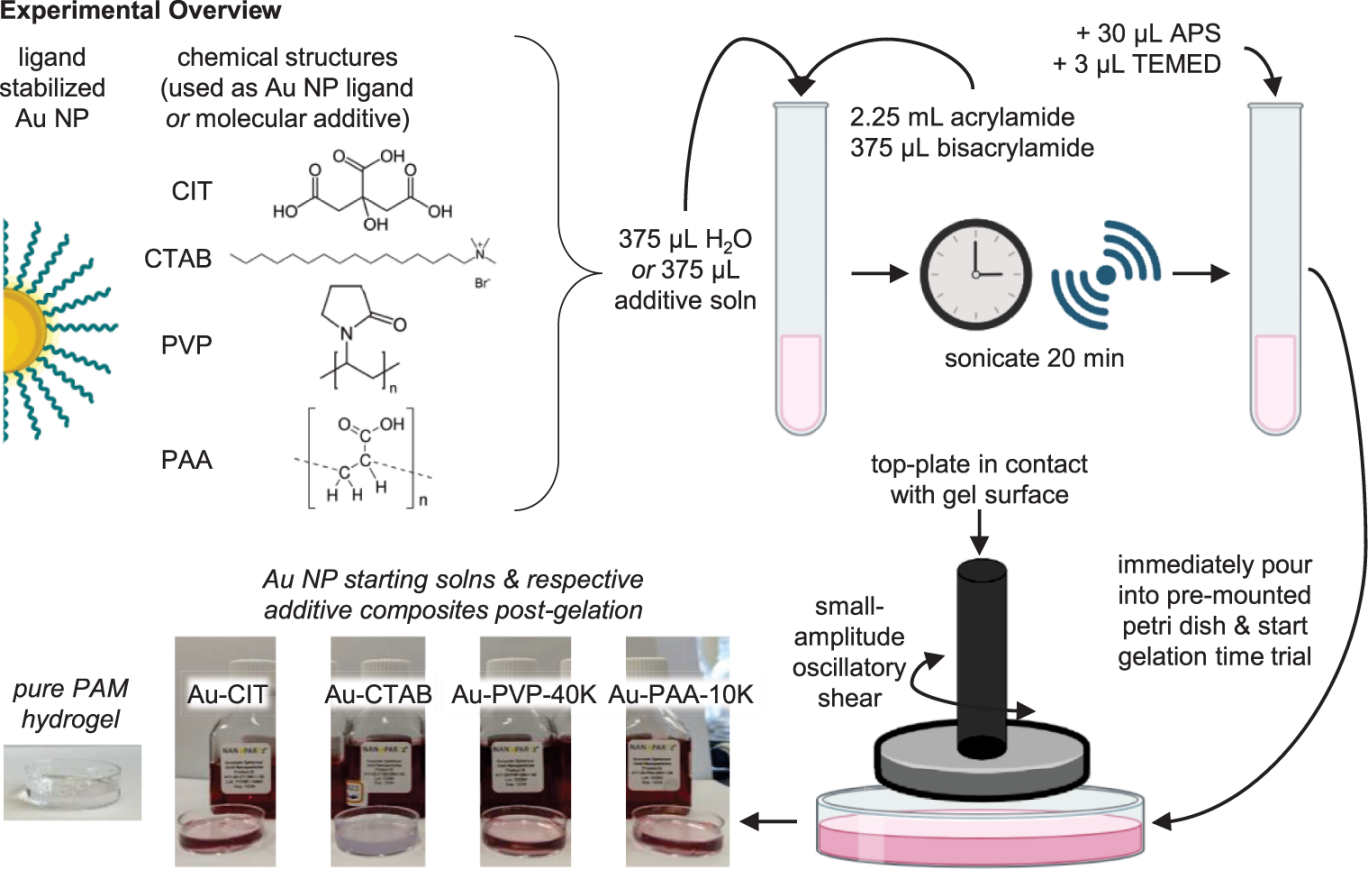
Schematic overview of the experimental gelation time process. An acrylamide and N,*N*′-methylenebisacrylamide (bisacrylamide) solution is made with either water or an additive solution and sonicated. Then the prepared APS and TEMED solutions are added, and the combined precursor solution poured immediately into a premounted Petri dish in the rheometer. Photos are included for visual reference of a pure PAM hydrogel and Au NP nanocomposite hydrogels. (Created in part with BioRender.com.).

For rheological time sweeps, the same quantities described above of acrylamide-based solution, TEMED, and APS were combined immediately preceding the start of a time trial. This was done by mixing the three solutions in a test tube, then immediately pouring the final mixed solution into a premounted Petri dish in the rheometer. For composite hydrogels the same processes were followed, using 375 μL of additive solution (at 0.05 mg/mL) to replace the 375 μL of water. This resulted in a final diluted concentration of 0.0062 mg/mL, or 6.2 ppm, for additives within the composite hydrogel. A schematic of the process is shown in Figure 1. The additive solutions are described in the following section.

### 2.2 Hydrogel Additives

For understanding the gelation of nanocomposite hydrogels in potential drug-delivery applications, gold nanoparticles (Au NPs) were used as representative nanomaterials with different capping ligands selected to examine the behavior of a range of chemical structures. Gold nanoparticles were used as received: Au NPs with a citrate (CIT) capping ligand were obtained from Sigma-Aldrich and Nanopartz, and Au NPs with cetyltrimethylammonium bromide (CTAB), polyvinylpyrrolidone (PVP, 40K g/mol), and poly(acrylic acid) (PAA, 10 K g/mol) capping ligands were obtained from Nanopartz. All NPs were 20 nm in diameter and stabilized with their respective capping ligands in Milli-Q water at an optical density (OD) of 1, or 0.05 mg/mL solution concentration. Manufacturer reported Zeta Charges (mV) were Au-CIT: −35; Au-CTAB: 35; Au-PVP-40K: −10; and Au-PAA-10K: −30. Additional gelation trials were done to examine the effect of Au NP concentration, using Au-CIT as a representative sample and diluting the 0.05 mg/mL solution in Milli-Q water to 75% (0.0375 mg/mL), 50% (0.025 mg/mL), and 25% (0.0125 mg/mL).

Molecular additives were prepared to help elucidate if the presence of the Au NPs influenced hydrogel properties, or if any impacts observed were strictly due to the chemistry of the capping ligand. Sodium citrate dihydrate (CIT), CTAB, PVP (average molecular weight 40 K g/mol), PAA monomer, and poly (acrylic acid sodium salt) (PAA, average molecular weight 5.1 K g/mol) were all obtained in powder form from Sigma-Aldrich. Solutions of each additive were made in Milli-Q water at 0.05 mg/mL concentration. Table 1 provides a summary of both types of additives, including the labels used for discussion in subsequent sections.

**Table 1:** Summary of capped nanoparticle (NP) additives and corresponding molecular additives^a^

**Additive**	**Capping Ligand**	**Molecule**	**Molecular Weight**(g/mol)	**Label**
Au NP	citrate	-	192	Au-CIT
	cetyltrimethylammonium bromide	-	364	Au-CTAB
	polyvinylpyrrolidone	-	40 K	Au-PVP-40K
	poly(acrylic acid)	-	10 K	Au-PAA-10K
molecular	-	sodium citrate dihydrate	294	CIT
	-	cetyltrimethylammonium bromide	364	CTAB
	-	polyvinylpyrrolidone	40 K	PVP-40K
	-	acrylic acid	72	PAA-72
	-	poly(acrylic acid)	5.1 K	PAA-5.1K

a All gold nanoparticles have a 20 nm manufacturer reported diameter and optical density, OD = 1. All additive solutions have a concentration of 0.05 mg/mL

### 2.3 Characterization

A suite of methods was used to characterize the additives and formed gels, described in detail below and data included in Supporting Information.

To assess the viscosity of the different additive solutions, parallel plate dynamic viscosity measurements were taken as a function of shear rate (HR 20 Discovery Hybrid Rheometer, TA Instruments). The shear rate was defined at the edge of the rotating plate, as the angular velocity multiplied by an instrument-dependent constant. For measurements, 1 mL of nanoparticle solution was slowly pipetted into a 2 mm gap while the top plate (20 mm diameter, steel) rotated at 1 rad/s. The gap was reduced to 1 mm under continuing rotation, and excess fluid trimmed. A 1 mm gap was maintained during the viscosity measurements. Figure S1 shows the viscosity curves for all samples as an average of three trials and error bars as the standard deviation between trials.

Nanoparticle additive solutions were characterized with dynamic light scattering (DLS), UV–visible (UV–vis) spectroscopy, and scanning electron microscopy (SEM). For DLS, the nanoparticle solutions were diluted by adding four to six drops to a cuvette filled to approximately 85% with Milli-Q water. Triplicate DLS measurements were taken in a Malvern Zetasizer Pro, using 0.20 as the refractive index and 2.43 as the absorption constant for Au.^41^ Particle size distribution curves are shown in Figure S2a. For UV–vis (Hitachi double beam U-2910 Spectrophotometer), undiluted nanoparticle solutions were measured as received, with spectra in Figure S2b. For SEM (JEOL JSM-IT700HR), 25 μL of each nanoparticle solution was deposited on carbon tape and dried under nitrogen gas. Images were taken at 5.0 kV. Figure S3 shows representative images.

Molecular additives were characterized as the as-received powder through attenuated total reflectance Fourier-transform infrared spectroscopy (ATR-FTIR, Thermo Scientific Nicolet iS50 FTIR Spectrometer). Spectra were collected as an average of 128 scans and 2 cm^–1^ resolution, with an air background collected before each sample. Each sample was normalized to the highest intensity peak in the 1800–700 cm^–1^ range, with spectra further offset in Figure S4 for clarity.

Postgelation time trials, composite hydrogel samples were characterized with confocal Raman microspectroscopy (Horiba XploRA PLUS). Spectra for the composite hydrogels were taken under a 10× objective using a 532 nm laser (10% power), 1800 gr/mm grating, and averaging 5 3-s accumulations over a 0–2500 cm^–1^ spectral range. Figure S5 highlights the 300–1700 cm^–1^ range for clarity, with normalization to the C–C backbone peak at ca. 1110 cm^–1^ and vertical offset.

### 2.4 Rheological Testing

Rheological tests were conducted at room temperature (20–22 °C) with an HR 20 Discovery Hybrid Rheometer, implementing a flat 20 mm diameter steel top plate. To monitor gelation times, a rheological protocol developed by Zuidema et al.^40^ was adapted. Strain and frequency sweeps on preformed polyacrylamide hydrogel were first taken to determine appropriate strain and frequency parameters for subsequent gelation time trials. For strain sweeps, the hydrogel storage modulus was measured as a function of percent strain under constant frequency (1 rad/s, or 0.16 Hz). For frequency sweeps, the hydrogel storage modulus was measured as a function of frequency under constant strain (1%). Discussed more in Section 3, a strain value of 1% and frequency of 10 rad/s (1.6 Hz) were used for gelation time trials.

Triplicate gelation trials (1% strain, 1.6 Hz) monitored storage modulus (*G*′) and loss modulus (*G*″) over time, starting from freshly mixed solution phase at 0 min and running up to 30 min, nominally at least 5× longer than the observed *G*′-*G″* crossover marking the gelation point.

## 3 Results and Discussion

A suite of characterization methods was employed to provide insight into the additives used to form composite polyacrylamide hydrogels, along with the samples postgelation trials. Discussed here, the corresponding data is summarized in Supporting Information. The first characterization, fluid viscosity, provided physical context for rheological gelation time measurements that are based on oscillations inherently sensitive to viscosity. Figure S1 shows the overlay of each additive solution, including pure Milli-Q water as a reference, with no discernible difference in fluid viscosity. This indicates that gelation times are not dependent on additive solution viscosity, and any differences observed are due to other chemical-physical properties.

Nanoparticle additives were measured in triplicate through dynamic light scattering (DLS) to determine if solution-phase size was impacting gelation time. Figure S2a shows a representative particle size distribution curve for each Au NP-ligand sample tested. In DLS, each sample deviated from the manufacturer reported 20 nm diameter, likely due to the measurement probing the hydrodynamic size and influence of the surrounding capping ligands. Au-CIT DLS diameter was 18 nm, Au-CTAB DLS diameter was 59 nm, Au-PVP-40K DLS diameter was 24 nm, and Au-PAA-10K DLS diameter was 24 nm. While Au-CTAB had an observed hydrodynamic size ca. 3x larger than the other Au NP additives, this was not reflected in additional UV–vis or SEM characterization. Moreover, UV–vis spectra (Figure S2b) for all four Au NPs indicated nanoparticle sizes more consistent with the manufacturer reported 20 nm diameter. All Au NP solutions had absorbances in the 518 to 522 nm range, with Au-CIT and Au-CTAB the minimum and maximum, respectively. Based on Haiss et al.,^42^ NP diameter can be determined by taking the ratio of absorbance at the surface plasmon resonance to the absorbance at 450 nm. The ratio for Au-CIT was 1.65, corresponding to a diameter of 16 nm. The ratio for Au-CTAB was 1.70, corresponding approximately to a 20 nm diameter (a ratio of 1.69 corresponds to 18 nm, and 1.73 corresponds to 20 nm).^42^ Comparing the UV–vis and DLS results, the larger observed diameter for Au-CTAB in DLS could be due to ligand effects, rather than NP aggregation. Additional SEM imaging (Figure S3) further supports consistent Au NP size. Most importantly, for the nanocomposite hydrogel formation discussed later, any differences in Au NP solutions do not appear to have a distinct impact on gelation times.

Routine chemical characterization of molecular structures was conducted to confirm basic functionality identification. Solution phase FTIR measurements of the molecular additives were attempted, however, no distinct signals for the additives were observed with the concentration likely below the detection limit of the instrument. To still confirm the chemical structure of the different molecular additives, ATR-FTIR spectra were collected for the as-received powders. Figure S4 highlights the characteristic peaks, and Table S1 provides peak assignments. Beyond the additives, Figure S5 provides Raman characterization of the composite hydrogels postgelation. Following gelation time trials, confocal Raman spectra were collected to confirm successful gelation with each sample exhibiting characteristic polyacrylamide peaks, detailed in Supporting Information. No peaks were observed for any of the individual Au NP ligands or molecular additives, but this is likely due to the low final concentration within the polyacrylamide hydrogel matrix. Under visual inspection, PAM hydrogels with the Au NP based additives had a light red wine color characteristic of 20 nm Au NPs, with the exception of Au-CTAB.^43^ For Au-CTAB nanocomposite hydrogel, there is a noticeable change from the red wine starting solution to a hazier light purple gel color. Based on work from Li et al.,^44^ this could be due to the dilution in the gel synthesis process and associated concentration-dependent assembly behavior of CTAB on Au NPs, such as an incomplete monolayer. For the Au NP gels overall, while the coloring does not demonstrate uniform distribution within the matrix, it does informally confirm these additives were incorporated within the gel. Pictures of the colored Au NP gels are shown in Figure 1, highlighting the overall process and example Au NP gels formed.

**Figure 2:**
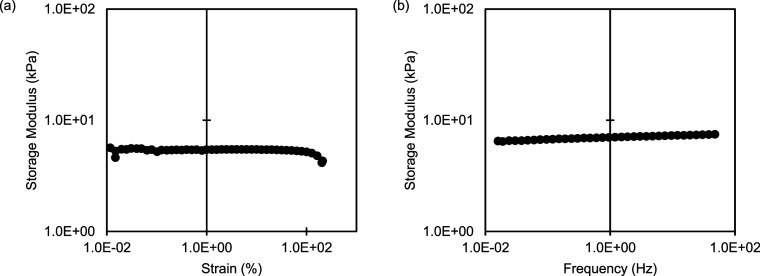
Representative strain and frequency sweeps taken on a preformed polyacrylamide hydrogel. (a) Strain sweep, taken at an angular frequency of 1 rad/s, or ca. 0.16 Hz. (b) Frequency sweep, taken at 1% strain.

To monitor the gelation of a hydrogel over time using small-amplitude oscillatory shear (SAOS), it is critical to use appropriate strain and frequency values. Preceding gelation time trials, such sweeps were conducted on a preformed polyacrylamide hydrogel. Shown in Figure 2a, the storage modulus was measured as oscillatory strain increased at constant frequency (1 rad/s). This identified the linear viscoelastic region (LVR) of the PAM hydrogel, where the modulus is independent of stress.^40,45^ A strain value of 1% is well within the LVR plateau, used for gelation time trials. A frequency sweep (Figure 2b) increases the rate of oscillation at constant strain—here, using 1% based on the prior strain sweep. The low frequency plateau is associated with the presence of a gel, allowing selection of a frequency value reflecting the formation of a gel network.^40^ Based on the continuous plateau for the PAM hydrogel, in this case all frequencies tested reflected gel behavior, thus a central value of 10 rad/s, 1.6 Hz, was implemented for gelation time trials.

**Figure 3:**
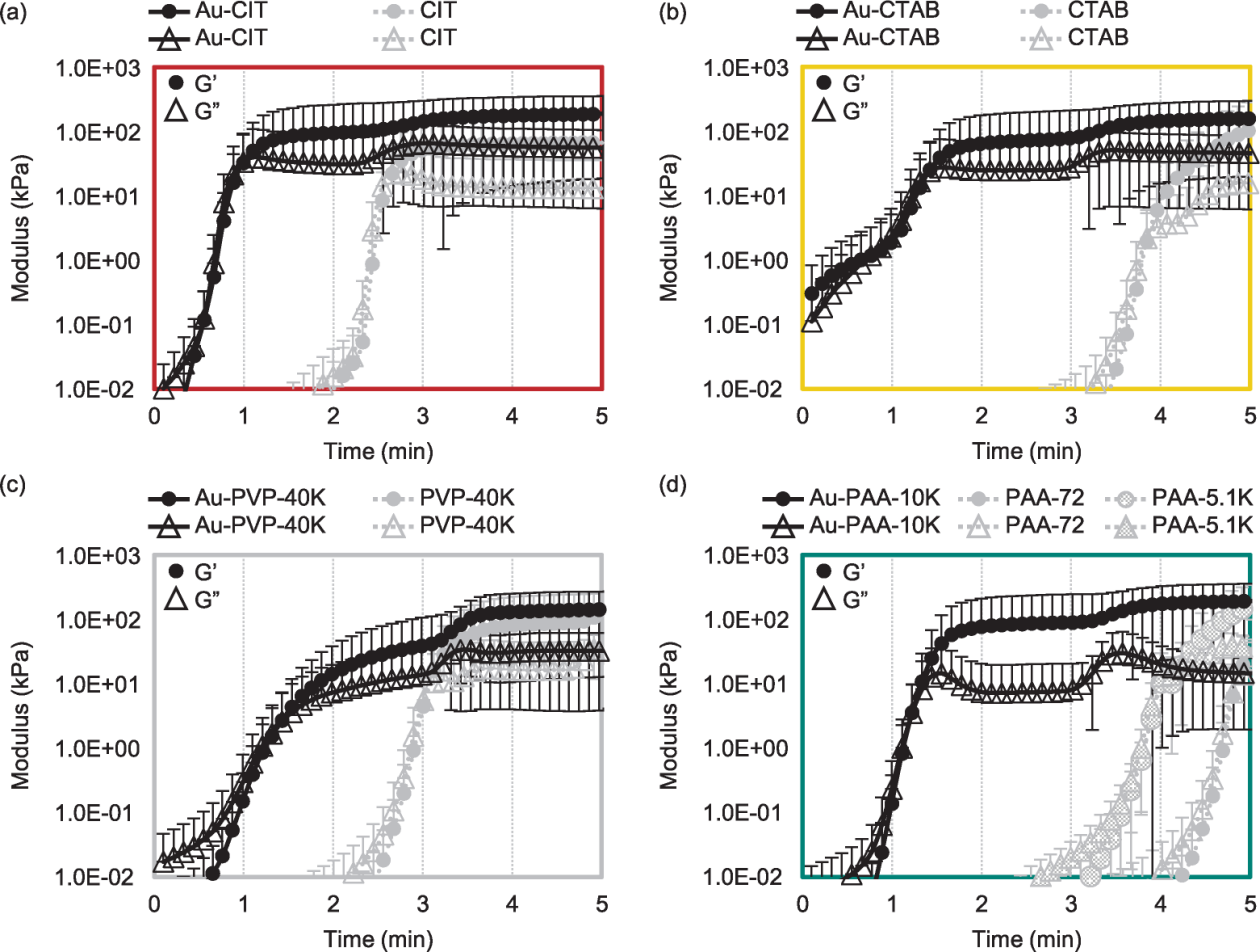
Time sweeps for each chemical structure tested, comparing the molecule as a Au NP capping ligand vs isolated molecular additive. Storage and loss moduli were measured as a function of time, at 1% strain and a frequency of 10 rad/s (ca. 1.6 Hz). Curves are plotted as the average of three trials, with error bars representing the standard deviation. For all data, circles indicate the storage modulus (*G*′) and triangles represent the loss modulus (*G″*). Solid black lines indicate Au NP additives, and gray dashed lines indicate molecular additives. The *G*′-*G″* crossover point dictates the gelation time, with (a) showing Au-CIT vs CIT, (b) Au-CTAB vs CTAB, (c) Au-PVP-40K vs PVP-40K, and (d) Au-PAA-10K vs PAA-72 and PAA-5.1K.

**Figure 4:**
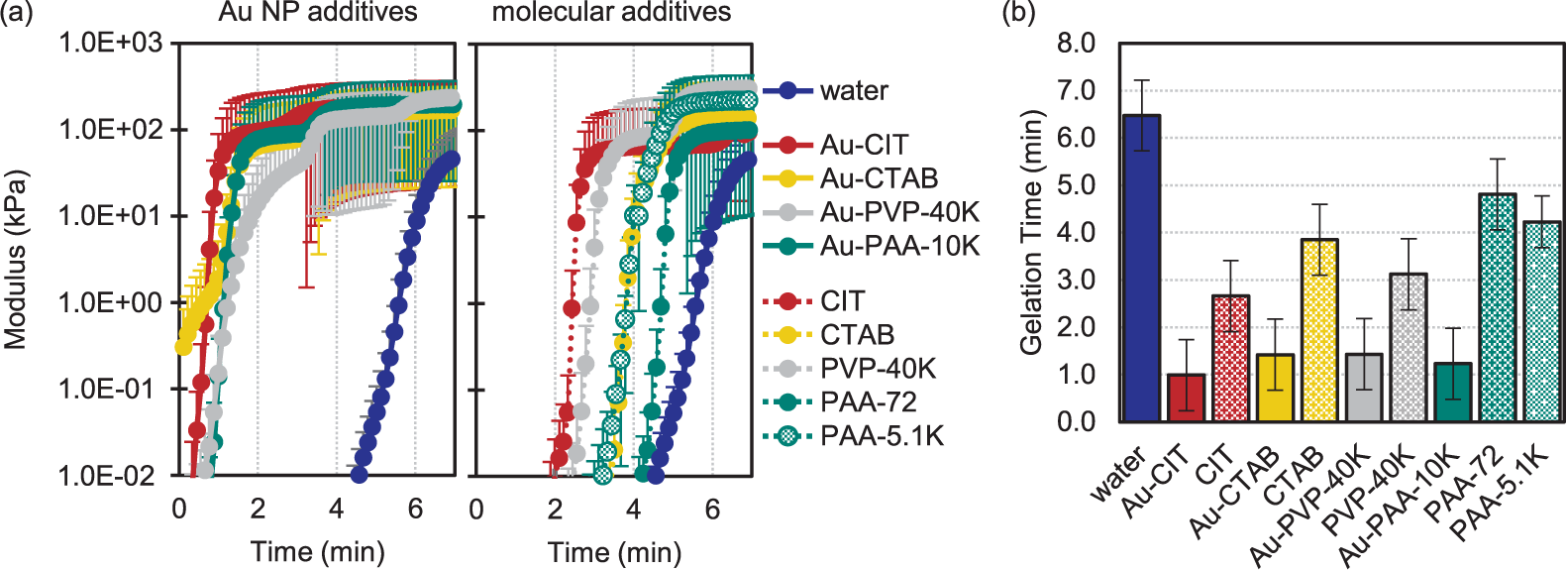
(a) Comparison of composite hydrogel gelation times grouped by additive type, Au NPs vs corresponding molecules. Note that this is the same data as in Figure 3, replotted to highlight differences between additive types, and only showing the storage moduli curves for clarity. Gelation times are accelerated to under 2 min for the Au NP nanocomposite hydrogels, regardless of ligand molecular structure. This is relative to “water,” the control sample of pure PAM hydrogel (no additive). Gelation times are also accelerated in the presence of the molecular additives, but not to the same extent as for the Au NP additives, and with slight differences in gelation time based on molecular structure. (b) Bar graph of average gelation times. The “water” sample is for a pure PAM hydrogel with no additives. The remaining samples are a side-by-side comparison for each chemical structure tested, showing the accelerated gelation times for the Au NP additives, regardless of capping ligand.

Using these experimentally determined strain and frequency values, gelation time trials monitored the storage (*G*′) and loss (*G″*) moduli for each additive. Figure 3 shows the average *G*′ and *G″* curves of three trials, with error bars representing the standard deviation. The steady-state storage modulus averaged across all trials is 2.5 × 10^2^ ± 0.6 × 10^2^ kPa, with no trend or significant difference (outside of 1 standard deviation) between samples. Focusing then on early state dynamics, the results are grouped by chemical structure, comparing the gelation behavior as a Au NP capping ligand vs a stand-alone molecular additive. In these measurements, the gelation time is defined as the *G*′-*G″* crossover point.^45,46^ After this crossover gelation has occurred the storage modulus of the gel is higher than the loss modulus, marking the sol-to-gel transition from more fluid-like viscous behavior to more solid-like elastic behavior.^28^ For each comparison in Figure 3, the molecule used as a Au NP capping ligand has an accelerated gelation time relative to the stand-alone molecular additive. To help interpret this observation, the same data (highlighting the storage modulus) is replotted in Figure 4a, grouping the trials by Au NP vs molecular additive and including a “water” reference of pure PAM hydrogel (no additive). Further, the *G*′-*G″* crossover points were quantified and summarized in the bar graph in Figure 4b.

The gelation time range of 1.0 ± 0.8 min to 4.8 ± 0.8 min for composites (Figure 3) and 6.5 ± 0.7 min for pure PAM (included in Figure 4) is comparable to the scope of literature values. Multiple conditions impact other reported gelation times, including the amount of cross-linker and temperature for polyacrylamide. At room temperature (20–22 °C) polyacrylamide had a gelation point of <15 min.^47^ For broader context, other systems have gelation dynamics that range from 1.4 to 7.0 min for hyaluronic acid/carboxymethyl cellulose based hydrogels^48^ and <15 min for network forming solutions of polyethylene glycol with and without silica-coated nanocapsules,^28^ or longer (on the order of hours) when working with partially hydrolyzed polyacrylamide.^49^ In this context the results here, rather than demonstrating a distinct acceleration of gelation time relative to other systems, focus on understanding the influence of NP capping ligand chemistry.

Here, the *G*′-*G″* crossover points, or gelation times, corresponding to the bar graph in Figure 4b are water at 6.5 ± 0.7 min; Au-CIT: 1.0 ± 0.8 min; CIT: 2.7 ± 0.8 min; Au-CTAB: 1.4 ± 0.8 min; CTAB: 3.9 ± 0.8 min; Au-PVP-40K: 1.4 ± 0.8 min; PVP-40K: 3.1 ± 0.8 min; Au-PAA-10K: 1.2 ± 0.8 min; PAA-72:4.8 ± 0.8 min; PAA-5.1K: 4.2 ± 0.5 min. Overall, there is no significant difference in gelation time based on molecular structure, especially when used as capping ligands for the Au NPs. There is some separation in *G*′-*G″* crossover points when used as independent molecular additives, but not outside of the deviation observed between repeated trials. While not significant, the trend for molecular additives in order of slowest to fastest gelation times is water (slowest) < PAA-72 < PAA-5.1K < CTAB < PVP-40K < CIT (fastest). This is consistent with molecular weight dominating gelation times for the polymer-based molecules (PAA-72 < PAA-5.1K < PVP-40K), with higher molecular weight compounds having shorter gelation times.^32,49^ CTAB and CIT could be further influenced by charge or available reaction sites, including hydrogen bonding.

Focusing on the Au NP additives the gelation time is notably accelerated compared to the molecular additives. The *G*′-*G″* crossover point for all Au NP capping ligands is from 1.0 ± 0.8 min to 1.4 ± 0.8 min, whereas the fastest gelling molecular additive was CIT at 2.7 ± 0.8 min. To more broadly test statistical differences between additive type, the Au NPs and molecular additives were treated as two separate groups for a *t* test analysis (Case 2).^50^ The overall Au NP additive gelation time average and standard deviation were  = 1.27 min and *s*_1_ = 0.21 min, respectively (*n* = 4). For the molecular additives, the gelation time average was  = 3.73 min and standard deviation was *s*_2_ = 0.86 min (*n* = 5). An F-test yielded statistically significantly different standard deviations, with *F*_calculated_ (17.26, eq 1) > F_table_ (9.12 at the 95% confidence level).
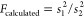
1

Using eq 2, *t*_calculated_ was 9.84, which was greater than t_table_ = 2.57 (at the 95% confidence level, using eq 3 to determine degrees of freedom).
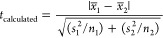
2
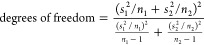
3

With *t*_calculated_ > t_table_, there is a statistically significant difference between the net gelation time of the Au NP additives from the molecular additives.

Considering the characterizations (Supporting Information) alongside the individual chemical structures, this indicates that for Au NP additives the gelation time is not impacted by viscosity (all additive solutions had the same viscosity), hydrodynamic particle size (DLS), Au NP size (all NPs had the same size by UV–vis), zeta potential (manufacturer reported values), or differences in chemical functionality, e.g., monomer vs polymer, molecular weight, linear vs ring structures, or extent of hydrogen bonding sites. The single dominating factor impacting composite polyacrylamide gelation times was whether or not Au NPs were present. Since temperature and concentration were also held constant, this suggests that the accelerated gelation times are due to the Au NPs acting as cross-linking agents within the PAM hydrogel, despite the different NP surface chemistries.

**Figure 5:**
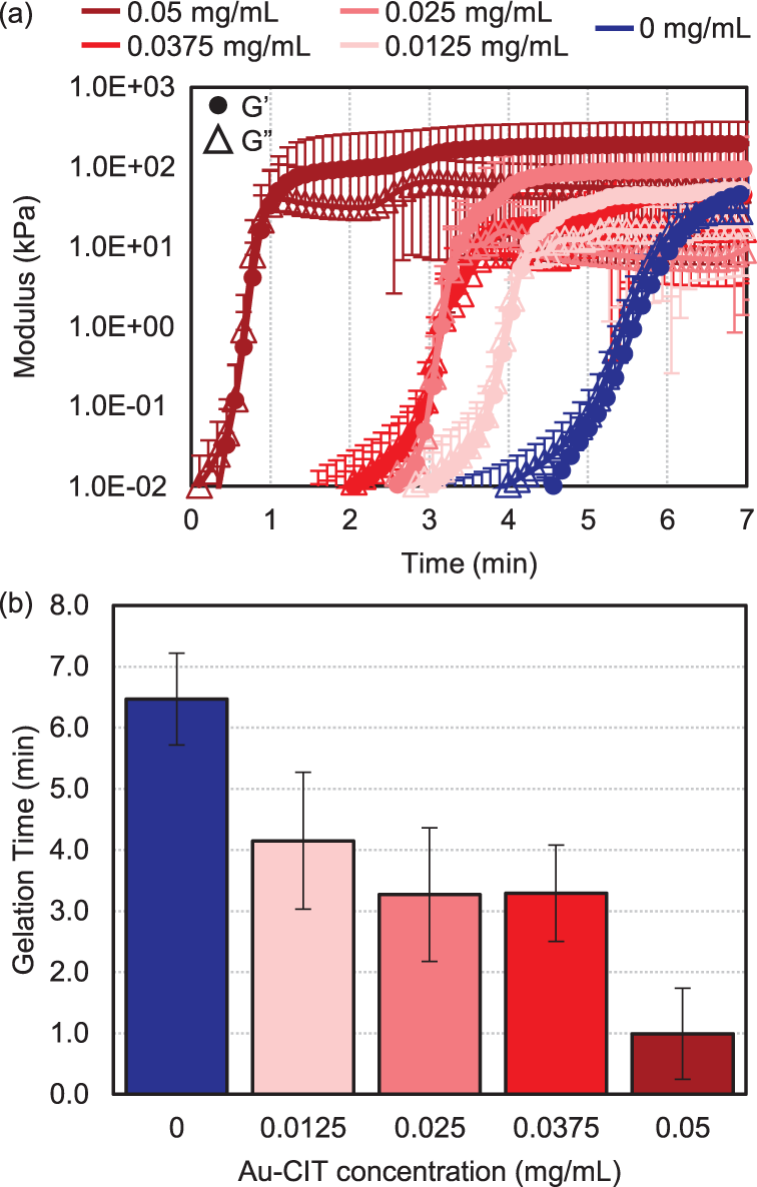
(a) Time sweeps for each Au-CIT concentration tested. As with the earlier discussed data, storage and loss moduli were measured as a function of time, at 1% strain and a frequency of 10 rad/s (ca. 1.6 Hz). Circles indicate the storage modulus (*G*′) and triangles represent the loss modulus (*G″*). Dark red indicates the original 0.05 mg/mL concentration, and lightening shades of red correspond to the decreasing concentrations. The blue curve is the pure PAM hydrogel, or 0 mg/mL (no Au-CIT added). (b) Bar graph of corresponding average gelation times.

To test if Au NPs act as cross-linkers, Au-CIT was used as a representative NP additive, with the greatest differential in gelation time from a pure PAM hydrogel. As described in Section 2.2, the original 0.05 mg/mL Au-CIT solution was diluted to 0.0375 mg/mL, 0.025 mg/mL, and 0.0125 mg/mL. Figure 5a shows the time sweeps for the original Au-CIT 0.05 mg/mL and pure PAM hydrogel in comparison to the new Au-CIT concentrations (as an average of three trials). The *G*′-*G*′′ crossover points for all of the samples shown are Au-CIT 0.05 mg/mL: 1.0 ± 0.8 min; Au-CIT 0.0375 mg/mL: 3.3 ± 1.1 min; Au-CIT 0.025 mg/mL: 3.3 ± 1.1 min; Au-CIT 0.0125 mg/mL: 4.2 ± 0.8 min; and pure PAM (0 mg/mL, or in Figure 4 noted as “water”): 6.5 ± 0.7 min. Figure 5b summarizes these gelation times in a bar graph.

There is not a statistically significant difference in gelation time for the intermediate Au-CIT concentrations tested, though a trend still emerged pointing toward gelation time decreasing with increasing Au NP concentration. Based on this, we suggest that it is feasible for the Au NPs to have cross-linking capacity within the gelation process.

An alternative mechanism is based on redox activity. In the four-part system (acrylamide monomer, bisacrylamide cross-linker, APS radical initiator, and TEMED redox catalyst), the Au NPs could contribute radicals, further catalyzing the gelation. Au NPs exhibit size dependent redox activity,^51,52^ and their electrochemical peak oxidation potentials are sensitive to the capping ligand used.^53^ As a cursory check here Au NP hydrogel gelation was tested by selectively removing one or both of APS and TEMED, replacing their volume with an equivalent amount of water. Using Au-CIT again as a representative NP, three altered hydrogel systems were made: 1) acrylamide/bisacrylamide/Au-CIT; 2) acrylamide/bisacrylamide/Au-CIT/APS; 3) acrylamide/bisacrylamide/Au-CIT/TEMED. These three mixtures did not gel, remaining liquid even after 72 h. This does not prove that Au NPs are not accelerating the gelation through redox catalysis, but the observation that in particular mixture 2 (acrylamide/bisacrylamide/Au-CIT/APS) did not gel indicates that any Au NP redox activity alone is not enough drive the reaction. In order to definitively assess Au NP redox activity as a potential gelation acceleration mechanism, additional experiments outside the scope of the work here would be needed.

Based on the preceding discussions, it is worth exploring ways in which Au NPs may be acting as a cross-linker. There is some evidence for Au to directly participate in hydrogen bonding,^54^ and acrylamide is an effective stabilizing solvent in the synthesis of Au NPs.^55^ For the system here, however, we suggest the most likely mechanism is due to Au NPs having a multivalent surface with the capacity to form multiple bonds within the gel networks.^56^ Given the independent behavior relative to capping ligand identity, we propose the underlying mechanism is a ligand exchange for a more energetically favorable Au–N interaction with the nitrogen in the polyacrylamide. Based on the binding energies of cetyltrimethylammonium (Au-CTA, −0.97 eV), trisodium citrate (Au-TSC, −0.90 eV), and polyvinylpyrrolidone (Au-VP, −1.07 eV), Fan et al.^57^ demonstrated diethylamine (Au-DEA, −1.33 eV) had the highest binding energy over the prior list of ligands due to the formation of strong Au–N bonds, experimentally facilitating ligand exchange. Dinkel et al.^58^ further described a fast vs slow ligand exchange process on Au NPs. The fast process (<100 s) was observed on low-coordinated Au sites, such as edges, with citrate readily replaced with 3-mercapto-1-propanesulfonate.^58^ Pertaining to thiol ligands, Cometto et al.^59^ detailed a range of methanethiol-gold binding energies based on molecular orientation and position on the surface structure, ranging from −27.81 to −42.36 kcal/mol, or −1.21 eV to −1.84 eV. The DEA binding energy from Fan et al.^57^ at −1.33 eV is on the low of the range of reported Au–S bond strengths, but in our case in the combined absence of a thiol ligand, relative binding energies, and experimental ligand exchange sequence demonstrated by Fan et al.^57^ support this as a possible mechanism. Determining the exact rates for the samples here would constitute a more in-depth separate body of work, but a rapid ligand exchange might explain the <2 min gelation times observed. It is also unclear at this stage if weaker ligands for Au accelerate gelation times relative to stronger bonds, and if smaller ligands are more easily displaced relative to multivalent polymeric ones, with these questions subjects for future work.

Overall, we find that in the presence of Au NPs the gelation time is rendered independent of the capping ligands examined, with the cross-linking rate likely dominated by the multivalency of Au and strength of the Au–N interaction. This supports the potential for Au NP-PAM nanocomposite hydrogels to be used as robust drug delivery vehicles in a diverse range of time sensitive biological systems or biomedical applications.

## 4 Conclusions

Using rheological time sweeps, the results here find that the prevailing factor impacting nanocomposite hydrogel gelation time is the presence of ligand-stabilized Au NPs. This is independent of the chemical nature of the capping ligand, having assessed a range of surface functionalities that may be found in biomedically relevant applications. Further, it is suggested that the accelerated gelation times rely on the Au NPs acting as a cross-linker promoting gelation, as the chemical structures incorporated as isolated molecular additives do not exhibit the same enhanced rate of gelation. With consistent gelation times, Au NP nanocomposite hydrogels have strong viability for use as therapeutics in the healthcare sector.

## Supporting Information Available

Additive and nanocomposite hydrogel characterization (PDF)
