# Supplementary material for: Accelerated Nanocomposite Hydrogel Gelation Times Independent of Gold Nanoparticle Ligand Functionality
Source: ACS Omega. 2024 Oct 14;9(42):42858–67. doi: 10.1021/acsomega.4c05102 (PMC11500131; doi:10.1021/acsomega.4c05102)
Supplement: Supplementary file 1 — ao4c05102_si_001.pdf [file ao4c05102_si_001.pdf]

Supporting Information:

Accelerated Nanocomposite Hydrogel Gelation Times Independent of Gold Nanoparticle Ligand Functionality

Brianna Couturier,[1] *Gloria Kozak,*[1] *John Levering,*[1] *Anna Zini,*[1] *Meagan B Elinski*[1*]

[1]Department of Chemistry, Hope College, Holland, Michigan 49423, United States

*E-mail: elinski@hope.edu

Supporting Information.

Discussed near the beginning of **Section 3** of the main text, with experimental detail described in **Section 2.3** of the main text, **Figures S1-S5** below provide the supporting data for additive and nanocomposite hydrogel characterization.

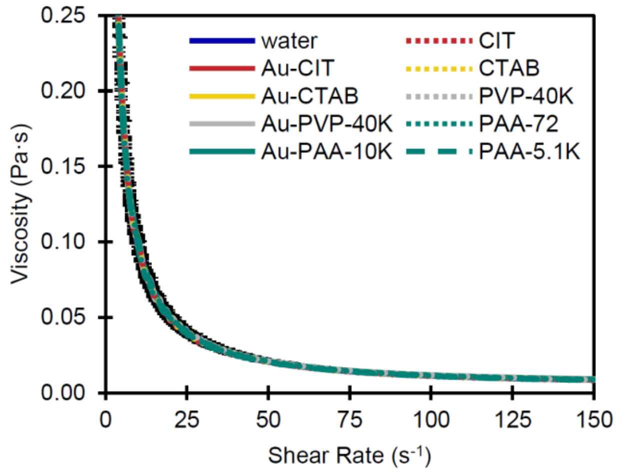

Figure S1. Parallel plate fluid viscosity measurements were taken as a function of shear rate for all additive solutions, with averages of three trials for each solution showing no difference from pure water.

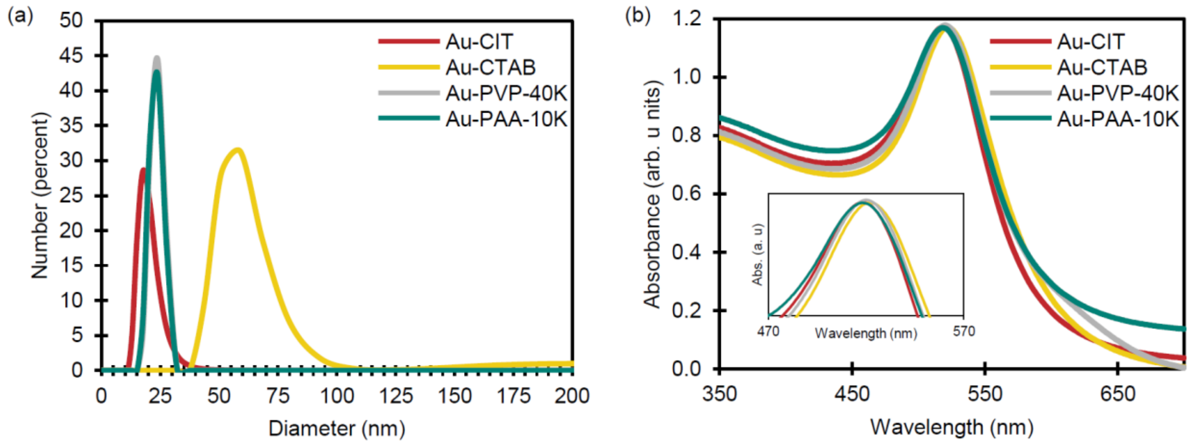

Figure S2. (a) Gold nanoparticle additives were characterized by dynamic light scattering, indicating the solution-phase particle size for each Au NP-capping ligand sample (measured in triplicate, representative curves shown). (b) UV-Vis spectra for each of the Au NP solutions showing nominal absorbance around 520 nm, typical of 20 nm diameter Au NPs. More detailed discussion in **Section 3** in the main text, with calculations based on the raw spectra. Here, the spectra have been vertically offset to highlight subtle shifts in the maximum absorption wavelength. The insert in (b) further zooms in on small shifts in the peak surface plasmon absorbance for each sample.

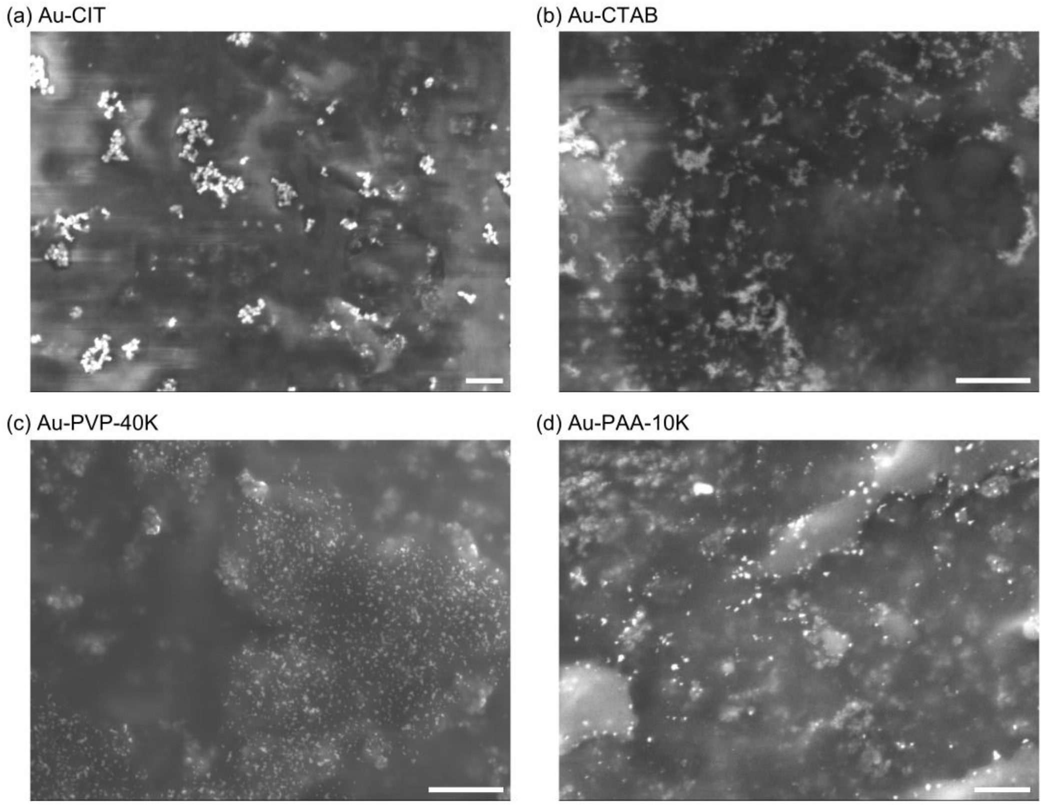

Figure S3. Example SEM images (secondary electron, at 5.0 kV) supporting the nanoparticle DLS and UV-Vis characterizations in **Figure S2**. Each solution was drop-cast onto carbon tape and dried under nitrogen: (a) Au-CIT, (b) Au-CTAB, (c) Au-PVP-40K, and (d) Au-PAA-10K. All scale bars are 1 µm.

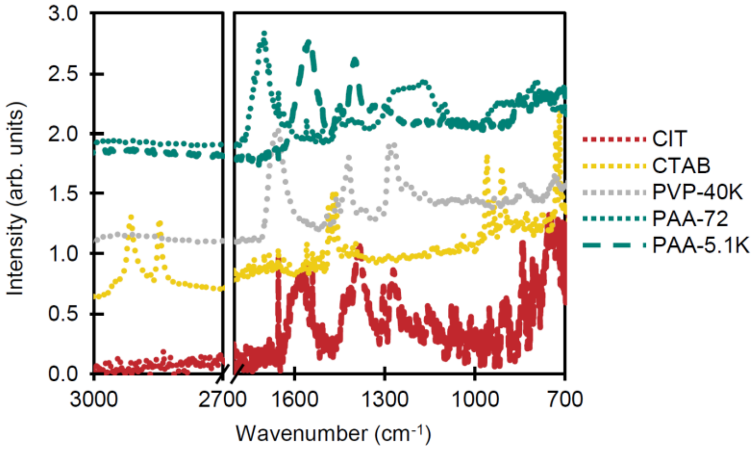

Figure S4. Molecular additives, in as-received powder form, were characterized with ATR-FTIR, confirming characteristic chemical functionalities for each respective structure. **Table S1** provides peak assignments.

Table S1. ATR-FTIR analysis of as-received powders of molecular additives, with peak assignments based on literature values for CIT,[1, 2] CTAB,[3-5] PVP-40K,[6-9] and PAA-72 (polyacrylic acid monomer),[10, 11] and PAA-5.1K.[10, 12]

Molecular Additive	Peak (cm^{-1})	Assignment
CIT	1572	C=O stretching
	1382, 1270, 1149	C-O stretching
	838, 771	C-H bending
CTAB	2915	asymmetric CH_2 stretch
	2848	symmetric CH_2 stretch
	1487	symmetric C-H scissoring of CH_3-N^+
	1472, 1461	CH_2 scissoring
	958	C-N stretching + C-C stretching + CH_3 rocking
	909	C-N stretching + CH_2 rocking
	730, 717	CH_2 rocking
PVP-40K	1652	C=O stretching
	1490, 1461, 1422	C-H deformations
	1371, 1314	C-H bending
	1286	C-N stretching
	845	pyrrolidone ring breathing
	725	C-C chain
PAA-72	1707	C=O stretching
	1462	CH_2 deformation
	1172	C-O stretching + O-H in-plane bending
	1109	C-CH_2 stretching
	799	CH_2 twisting + C-COOH stretching
PAA-5.1K	1555	C=O stretching
	1457	CH_2 deformation
	1401	C-O stretching + O-H in-plane bending
	1109	C-CH_2 stretching
	955	CH_2 rocking
	849	C-COOH stretching

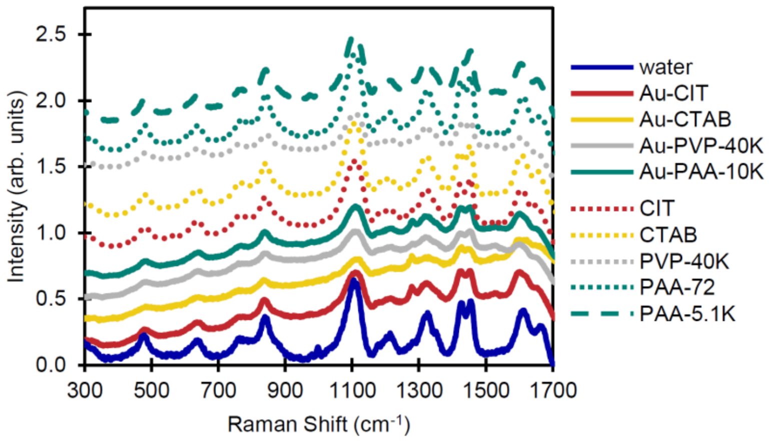

Figure S5. Representative Raman spectra for each nanocomposite hydrogel taken post-gelation time trials, confirming the formation of polyacrylamide-based hydrogels.

In **Figure S5**, each sample exhibits polyacrylamide peaks at 480 cm^{-1} (C-C deformation), 640 cm^{-1} (CH_2 torsion), 765 cm^{-1} (C-H wagging), 845 cm^{-1} (C-C symmetric stretch), 1000 cm^{-1} (low intensity, C-C asymmetric stretch), 1110 cm^{-1} (symmetric stretch), 1215 cm^{-1} (NH_2 wagging),

1330 cm^{-1} (C-H deformation), 1430 cm^{-1} (C-N stretching), 1460 cm^{-1} (CH_2 deformation), 1615 cm^{-1} (NH_2 deformation), and 1670 cm^{-1} (C-O stretching).[13-16]

References.

1. Thottoli, A. K.; Unni, A. K. A., Effect of Trisodium Citrate Concentration on the Particle Growth of ZnS Nanoparticles. *Journal of Nanostructure in Chemistry* **2013,** *3*, 56. DOI:10.1186/2193-8865-3-56
2. Mohan, J. C.; Praveen, G.; Chennazhi, K. P.; Jayakumar, R.; Nair, S. V., Functionalised Gold Nanoparticles for Selective Induction of In Vitro Apoptosis Among Human Cancer Cell Lines. *Journal of Experimental Nanoscience* **2013,** *8*, 32-45. DOI:10.1080/17458080.2011.557841
3. Gokce, H.; Bahceli, S., The Molecular Structures, Vibrational Spectroscopies (FT-IR and Raman) and Quantum Chemical Calculations of n-Alkyltrimethylammonium Bromides. *Optics and Spectroscopy* **2013,** *115*, 632-644. DOI:10.1134/S0030400X13110076
4. Xu, Y.; Huang, C.; Dang, X.; Khan, M. R.; Huang, H.; Zhao, Y.; Wang, S., Preparation of Long-Term Antibacterial SiO2-Cinnameldehyde Microcapsule via Sol-Gel Approach as a Functional Additive for PBAT Film. *Processes* **2020,** *8*, 897. DOI:10.3390/pr8080897
5. Gutierrez-Becerra, A.; Barcena-Soto, M.; Soto, V.; Arellano-Ceja, J.; Casillas, N.; Prevost, S.; Noirez, L.; Gradzielski, M.; Escalante, J. I., Structure of Reverse Microemulsion-Templated Metal Hexacyanoferrate Nanoparticles. *Nanoscale Research Letters* **2012,** *7*, 83. DOI:10.1186/1556-276X-7-83
6. Song, Y. J.; Wang, M.; Zhang, X. Y.; Wu, J. Y.; Zhang, T., Investigation on the Role of the Molecular Weight of Polyvinyl Pyrrolidone in the Shape Control of High-Yield Silver Nanospheres and Nanowires. *Nanoscale Research letters* **2014,** *9*, 17. DOI:10.1186/1556-276X-9-17
7. Mireles, L. K.; Wu, M. R.; Saadeh, N.; Yahia, L.; Sacher, E., Physicochemical Characterization of Polyvinyl Pyrrolidone: A Tale of Two Polyvinyl Pyrrolidones. *ACS Omega* **2020,** *5*, 30461-30467. DOI:10.1021/acsomega.0c04010
8. Borodko, Y.; Habas, S. E.; Koebel, M.; Yang, P.; Frei, H.; Samorjai, G. A., Probing the Interaction of Poly(vinylpyrrolidone) with Platinum Nanocrystals by UV-Raman and FTIR. *Journal of Physical Chemistry B* **2006,** *110*, 23052-23059. DOI:10.1021/jp063338+
9. Huang, S. W.; Lin, Y. F.; Li, Y. X.; Hu, C. C.; Chiu, T. C., Synthesis of Fluorescent Carbon Dots as Selective and Sensitive Probes for Cupric Ions and Cell Imaging. *Molecules* **2019,** *24*, 1785. DOI:10.3390/molecules24091785
10. Dong, J.; Ozaki, Y.; Nakashima, K., Infrared, Raman, and Near-Infrared Spectroscopic Evidence for the Coexistence of Various Hydrogen-Bond Forms in Poly(acrylid acid). *Macromolecules* **1997,** *30*, 1111-1117. DOI:10.1021/ma960693x
11. Kohestanian, M.; Bouhendi, H.; Keshavarzi, N.; Mahmoudi, M.; Pourjavadi, A.; Ghiass, M., Preparation of Poly(acrylic acid) Microgels by Alcohol Type Cross-Linkers and a Comparison with Other Cross-Linking Methods. *Polymer Bulletin* **2022,** *79*, 7775-7794. DOI:10.1007/s00289-021-03878-5
12. Alvarez-Gayosso, C.; Canseco, M. A.; Estrada, R. F.; Palacios-Alquisira, J.; Hinojosa, J.; Castano, V. M., Preparation and Microstructure of Cobalt(III) Poly(acrylate) Hybrid Materials. *International Journal of Basic and Applied Science* **2015,** *4*, 255-263. DOI:10.14419/ijbas.v4i3.4172
13. Murugan, R.; Mohan, S.; Bigotto, A., FTIR and Polarised Raman Spectra of Acrylamide and Polyacrylamide. *Journal of the Korean Physical Society* **1998,** *32*, 505-512.
14. Corona-Rivera, M. A.; Ovando-Medina, V. M.; Bernal-Jacome, L. A.; Cervantes-Gonzalez, E.; Antonio-Carmona, I. D.; Davila-Guzman, N. E., Remazol Red Dye Removal Using Poly(Acrylamide-co-Acrylic Acid) Hydrogels and Water Absorbency Studies. *Colloid and Polymer Science* **2017,** *295*, 227-236. http://dx.doi.org/DOI:10.1007/s00396-016-3996-2
15. Gyarmati, B.; Nemethy, A.; Szilagyi, Reversible Disfulphide Formation in Polymer Networks: A Versatile Functional Group from Synthesis to Applications. *European Polymer Journal* **2013,** *49*, 1268-1286. DOI:10.1016/j.eurpolymj.2013.03.001
16. Malkovskiy, A. V.; Tom, A.; Joubert, L.-M.; Bao, Z., Visualization of the Distribution of Covalently Cross-Linked Hydrogels in CLARITY Brain-Polymer Hybrids for Different Monomer Concentrations. *Scientific Reports* **2022,** *12*, 13549. DOI:10.1038/s41598-022-17687-x
